# Trichinellosis Outbreak

**DOI:** 10.3201/eid1112.050461

**Published:** 2005-12

**Authors:** Esther Marva, Alex Markovics, Michael Gdalevich, Nehama Asor, Chantal Sadik, Alex Leventhal

**Affiliations:** * Ministry of Health, Jerusalem, Israel; † Kimron Veterinary Institute, Beit-Dagan, Israel

**Keywords:** Trichinellosis, epidemiology, outbreak, serology, Israel, letter

**To the Editor:** Trichinellosis is a zoonotic disease caused by the nematode *Trichinella*. Although now uncommon as a result of public health control measures, trichinellosis outbreaks have been reported in the United States (*1*), Europe (*2**,**3*), Mexico (*4*), Thailand (*5*), Canada (*6*), Lebanon (*7**–**10*), and elsewhere.

In Israel, the disease is rare because most Jewish and Muslim citizens avoid eating pork. Until 1997, only 6 small outbreaks were reported in humans; they occurred mostly in the Christian Arab population. However, from 1998 to 2004, 10 similar trichinellosis outbreaks involving 200 Thai migrant agricultural workers occurred. The workers all took part in festive meals whose main dish was uninspected wild boar, hunted in the Upper Galilee in northern Israel, near the Lebanese border. Wild boar was also the source of several large outbreaks that were reported from 1975 to 1997 in southern Lebanon (*7**–**10*).

We report an outbreak among a group of 47 male Thai workers (mean age 32 years). The workers participated in a festive meal where the implicated wild boar meat was served. Two weeks later, 26 of them had symptoms of trichinellosis. Serologic tests were performed on all 47 workers 2–4 weeks after they ate the infected meat (first time point), 6 and 8 weeks later (second time point), or both. The specimens were tested for immunoglobulin G antibodies to *Trichinella spiralis* with the LMD Elisa kit lot 9910231 (Alexon-Trend, Ramsey, MN, USA). According to the kit insert, absorbance readings >0.3 optical density (OD) units are positive.

A case-patient was defined as a worker who had >1 of the following symptoms of trichinellosis: muscle soreness, edema of upper eyelids, fever, ocular symptoms, gastrointestinal symptoms, maculopapular rash, or pulmonary symptoms. Workers with no clinical symptoms were divided into 2 subgroups. Asymptomatic case-patients were workers with >1 positive serologic test result with or without elevated absolute eosinophil count. Nonpatients were workers whose serologic results remained negative during the 2 months of study, with normal absolute eosinophil count.

At the onset of symptoms, 2 weeks after the meal, 26 patients arrived at the emergency room of Barzilai Hospital, Ashkelon, with abdominal pain with various degrees of myalgia (23 [88%]), fever (3 [11%]), periorbital edema (11 [42%]), headache (12 [46%]), rash (9 [34%]), and cough (1 [4%]). Only 1 patient did not seroconvert during the 2-month study.

Of 18 symptomatic patients, 13 (72%) were positive at the first time point (mean ± standard deviation [SD] OD 0.87± 0.80; in another 4 patients, seroconversion was observed at the second time point. At this second time point, 21 persons were tested, and 20 (95%) were positive (OD 2.89 ± 1.16). Five patients showed moderate eosinophilia (1.0–5.0 × 10^9^ cells/L), and 4 patients had marked eosinophilia (>5.0 × 10^9^ cells/L). No direct correlation was observed between severity of symptoms, degree of eosinophilia, and antibody levels (OD).

Of the 21 asymptomatic workers, 7 did not have cases of trichinellosis, and 14 (67%) had >1 positive sample. At the first time point, 12 workers were tested; 7 (58%) were positive (OD 0.64 ± 0.91). At the second time point, seroconversion was observed in 4 other workers. At this time, 14 persons were tested; 10 (71%) were positive (OD 1.76 ± 1.62). In this group, 1 person had moderate eosinophilia, and 2 had marked eosinophilia.

All the persons who ate the infected meat were treated with mebendazole, 5 mg/kg twice a day for 5 days. All symptomatic patients recovered. Epidemiologic investigation indicated that 1 large piece of meat was put in boiling water for just a few minutes before being eaten. The meat that remained from the meal was examined microscopically, and encysted *Trichinella* larvae were identified (Figure).

**Figure:**
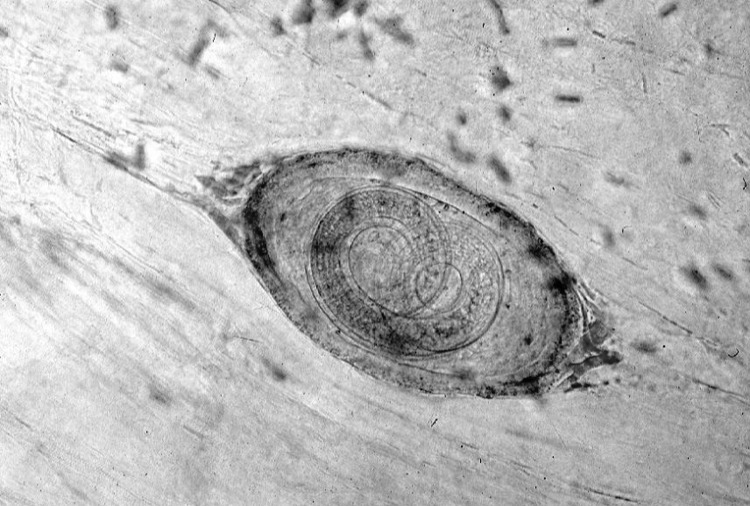
Trichinella larvae in a sample of infected meat (light microscopy, ×100).

The attack rate in this outbreak was higher (85%) than that in other published outbreaks. One explanation for this high rate could be that our case definition was broader and included any exposed person who had a positive serologic result during the 2-month study period. Moreover, all those who ate the investigated meal gave at least 1 blood sample. In other outbreaks, only samples from acute symptomatic patients were taken (*8*), the follow-up was incomplete because some patients did not return for convalescent-phase serologic testing (*8*), or not all the affected persons were studied (*7*).

This outbreak demonstrates the need to increase awareness and knowledge of trichinellosis and its epidemiologic features among medical personnel, public health teams, and workers. Health education and promotion are important for migrant workers, who should be reached and informed about how to prevent trichinellosis.
